# Immunoassays for scarce tumour-antigens in exosomes: detection of the human NKG2D-Ligand, MICA, in tetraspanin-containing nanovesicles from melanoma

**DOI:** 10.1186/s12951-018-0372-z

**Published:** 2018-05-02

**Authors:** Sheila López-Cobo, Carmen Campos-Silva, Amanda Moyano, Myriam Oliveira-Rodríguez, Annette Paschen, María Yáñez-Mó, María Carmen Blanco-López, Mar Valés-Gómez

**Affiliations:** 1 Department of Immunology and Oncology, National Centre for Biotechnology, CNB-CSIC, Madrid, Spain; 2 Department of Physical and Analytical Chemistry, University of Oviedo, Asturias, Spain; 3 Department of Dermatology, University Hospital Essen, University Duisburg-Essen, and German Cancer Consortium (DKTK) Partner Site Essen/Düsseldorf, 45122 Essen, Germany; 4 Department of Molecular Biology, UAM, Madrid, Spain; 5 Centro de Biología Molecular Severo Ochoa (CBM-SO), Instituto de Investigación Sanitaria Princesa (IIS-IP), Madrid, Spain

**Keywords:** Lateral flow, Immune capture, Exosomes, Tumour antigens, Steric hindrance, Aggregation

## Abstract

**Background:**

Tumour-derived exosomes can be released to serum and provide information on the features of the malignancy, however, in order to perform systematic studies in biological samples, faster diagnostic techniques are needed, especially for detection of low abundance proteins. Most human cancer cells are positive for at least one ligand for the activating immune receptor NKG2D and the presence in plasma of NKG2D-ligands can be associated with prognosis.

**Methods:**

Using MICA as example of a tumour-derived antigen, endogenously expressed in metastatic melanoma and recruited to exosomes, we have developed two immunocapture-based assays for detection of different epitopes in nanovesicles. Although both techniques, enzyme-linked immunosorbent assay (ELISA) and Lateral flow immunoassays (LFIA) have the same theoretical basis, that is, using capture and detection antibodies for a colorimetric read-out, analysis of exosome-bound proteins poses methodological problems that do not occur when these techniques are used for detection of soluble molecules, due to the presence of multiple epitopes on the vesicle.

**Results:**

Here we demonstrate that, in ELISA, the signal obtained was directly proportional to the amount of epitopes per exosome. In LFIA, the amount of detection antibody immobilized in Au-nanoparticles needs to be low for efficient detection, otherwise steric hindrance results in lower signal. We describe the conditions for detection of MICA in exosomes and prove, for the first time using both techniques, the co-existence in one vesicle of exosomal markers (the tetraspanins CD9, CD63 and CD81) and an endogenously expressed tumour-derived antigen. The study also reveals that scarce proteins can be used as targets for detection antibody in LFIA with a better result than very abundant proteins and that the conditions can be optimized for detection of the protein in plasma.

**Conclusions:**

These results open the possibility of analyzing biological samples for the presence of tumour-derived exosomes using high throughput techniques.

**Electronic supplementary material:**

The online version of this article (10.1186/s12951-018-0372-z) contains supplementary material, which is available to authorized users.

## Background

Normal cells release a variety of small sized vesicles, called extracellular vesicles (EVs) with different origins, size and composition [1–3]. Exosomes are a subtype of EVs that originate after fusion of subcellular organelles with the plasma membrane and release of the small intraluminal vesicles. These vesicles usually have a diameter of 50–150 nm and are enriched in markers from the endocytic pathway and tetraspanins such as CD63, CD9 and CD81. Exosomes carry different types of molecules including nucleic acids and proteins that can transfer information between different cells. Specific molecules are actively recruited to exosomes using different targeting methods, for example, miRNAs are included in exosomes when they carry a particular motif [4, 5] and proteins with a fatty acid or GPI modification are also more commonly found in these vesicles [6, 7]. The particular lipid composition of the exosome membrane may also contribute to the recruitment of specific biomolecules [8].

Exosomes can be found in many biological fluids, like plasma, urine, saliva and interstitial liquid of healthy donors and patients, but their roles in human physiology are largely unknown [9]. However, because only certain cellular molecules are incorporated into exosomes, analysis of the composition of these nanovesicles can provide significant information about the cell of origin. Thus, there is great interest in developing methods for the detection of biomolecules contained in exosomes and to explore the possibility of using them as a tool for diagnosis.

Exosomes are released in large quantities from certain tumour cells and tumour-derived exosomes can be detected in patient biological fluids [10], therefore, their analysis can provide information about the cell that originated the vesicles. For example, exosomes from melanoma cell lines contain tumour antigens such as Mart-1/MelanA [11–13]. In addition, tumour-derived exosomes have been also shown to contain molecules with immune modulation properties, such as apoptosis induction by FasL, TRAIL, PD-L1 [14], immune suppression through TGFβ [15], or antigen cross-presentation [11, 13].

A paradigm of a molecule released by tumour cells with immune modulation functions is MICA (MHC class I chain-related protein A), a protein belonging to stress-related molecules that bind to the activating immune receptor NKG2D (activating natural killer group 2D) expressed on all human NK and CD8^+^ T cells [16]. Soluble ligands for NKG2D, including MICA, have been found in serum from several types of cancer patients and, in humans, high levels of soluble NKG2D ligands generally correlate with higher tumor grade and worse prognosis [17]. Indeed, it has recently been shown that the presence of soluble NKG2D-ligands influences the clinical outcome of cancer patients treated with checkpoint-inhibitor monoclonal antibodies [18]. Similarly, the use of antibodies against the α3 domain of MICA to decrease the release of ligand also promotes tumour immunity [19]. We have previously described that certain NKG2D-ligands are recruited into exosome fractions and that they are potent modulators of the immune response [20, 21]. In fact, membrane-bound NKG2D-ligands downmodulate the receptor more efficiently than metalloprotease cleaved soluble species [22, 23]. Thus, it is very likely that the study of NKG2D-ligands contained in exosomes of biological fluids will shed light on the modulation of the immune response in cancer patients.

Since current protocols for exosome analysis often require expensive equipment and specialist knowledge [3], we aimed to develop new tools for detection of the tumour-derived protein MICA in exosomes, using methods based on immunocapture of tetraspanins, as general exosome markers, coupled with detection of MICA. Since different exosome populations can have different content of tetraspanins [24], we first defined different antibody combinations, for either detection or capture of vesicles, that could be used in enzyme-linked immunosorbent assay (ELISA), an immunological method that firstly immobilises the target antigen using a capture antibody followed by detection using a second, labelled-antibody. Using this technique, we demonstrate here for the first time the presence of MICA in vesicles containing CD9, CD63 or CD81.

We have previously shown that exosomes can be detected in lateral flow immunoassays (LFIA) using combinations of tetraspanin-specific mAbs [25]. This technique has the same conceptual basis as ELISA, however, in a first step, samples are mixed with detection antibody, previously attached to gold nanoparticles (AuNP) for visualization, and then the mixture is allowed to reach by capillarity a line of capture antibody recognizing a different epitope. While optimizing LFIA for detection of MICA-containing exosomes, we have discovered that the difference in the experimental set up between ELISA and LFIA can markedly affect the outcome of the experiment, because exosomes contain multiple epitopes of each target per vesicle both for capture and detection antibodies, and this can affect multimerization and aggregation. In fact, we demonstrate that abundant proteins should not be used as targets for a detection antibody in LFIA because, in this case, steric impediments on the limited surface area of exosomes can impair binding of the second capture antibody.

## Results

### Melanoma-derived exosomes express the endogenous stress-related molecule MICA

**Fig. 1:**
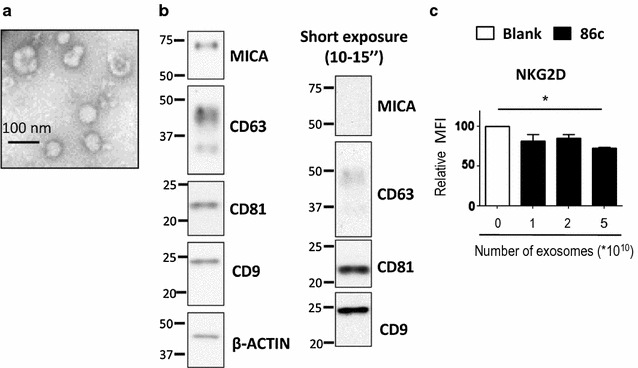
Characterization of exosomes from metastatic melanoma cell lines. Exosomes from Ma-Mel-86c cells were purified from tissue culture supernatant by ultracentrifugation. **a** TEM. Exosomes were analyzed by electron microscopy. Bar: 100 nm. **b** Western blot. MICA and tetraspanins (CD63, CD81 and CD9) were analysed by Western blot. Actin was used as loading control. The result from a representative experiment is shown. An exposure of 10–15 s is shown in the right panel. **c** NKG2D downmodulation. Activated NK cells were co-incubated with increasing amounts of Ma-Mel-86c exosomes as indicated. Cells were stained with anti-NKG2D and analysed by flow cytometry. The plot represents the change in the mean fluorescence intensity (MFI) of NKG2D on NK cells after incubation with exosomes, related to NK cells incubated in medium alone. Data are the mean and SEM obtained in three experiments using NK cells from different donors (*P < 0.05)

As a first step in the development of new antibody combinations to detect and analyse the composition of tumour-derived exosomes, nanovesicles isolated from tissue culture supernatant of the metastatic melanoma cell line, Ma-Mel-86c, were characterized. Nanosight analysis revealed that the size of the melanoma-derived exosomes was on average around 120 nm and this was confirmed by electron microscopy (Fig. 1a). The expression of widely expressed exosome markers, such as the tetraspanins CD9, CD81 and CD63, was verified by Western blot in the preparation of exosomes from the same cell line, Ma-Mel-86c (Fig. 1b). We have previously described that the metastatic melanoma cell line Ma-Mel-86c expresses the NKG2D-ligand MICA at the cell surface and that the products of particular alleles of this gene are found in fractions in which exosome markers were also detected [7, 26]. As shown, endogenous MICA was present in melanoma-derived nanovesicles, but was much less abundant than tetraspanins. Nevertheless, when melanoma-derived exosomes were co-cultured with NK cells, the NKG2D receptor was downmodulated from NK cell surface, demonstrating that endogenously expressed MICA is exposed on the exosome surface and that it is biologically functional (Fig. 1c).

### MICA can be detected by ELISA on tumour-derived exosomes

**Fig. 2:**
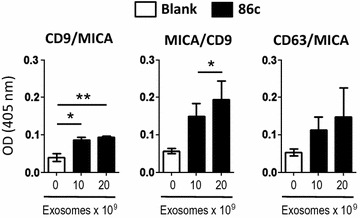
Detection of MICA-containing exosomes by ELISA, using different tetraspanin-specific antibodies. Different amounts of metastatic melanoma Ma-Mel-86c (86c) derived exosomes were analyzed by ELISA using the combinations of capture/detection antibodies indicated at the top of each plot (capture/detection). The plots represent the optical density (OD) at 405 nm. Negative controls (Blank) include capture and detection antibodies but no exosomes. Data are the mean and SEM of three independent experiments run in duplicates (*P < 0.05, **P < 0.01)

Once the exosomes from the melanoma cell line Ma-Mel-86c had been characterized phenotypically and functionally, the expression of MICA within these nanovesicles was analysed by ELISA. It is important to remember here that exosomes are heterogeneous in their protein composition and that WB analyses can only demonstrate co-fractionation of two markers, but cannot provide evidence for the coexistence of two proteins in the same particle. So, to prove that tetraspanins and MICA are present in the same vesicle, ELISA experiments were performed using combinations of antibodies against the different molecules (Fig. 2). Capture with anti-MICA and detection with anti-CD9 was a very efficient combination for an ELISA platform. The opposite orientation, i.e. capture with tetraspanin (CD9 or CD63) and detection with anti-MICA also provided positive readings although the sensitivity was clearly less. Since the amount of MICA is relatively low in these exosomes, when compared to the amount of tetraspanin, it seems likely that the capture of the exosome through MICA allows a better amplification of the readout signal detecting a very abundant tetraspanin like CD9.

### Exosomal MICA can be detected by LFIA

Next, since we have already demonstrated that exosomes can be detected using LFIA devices [25], experiments to choose the best antibody combination for the development of a new LFIA tool for the detection of MICA in exosomes were performed (Table 1 and Additional file 1: Figure 1A). MICA expression on melanoma-derived exosomes could be detected by LFIA using anti-CD9 for capture and anti-MICA for detection. Surprisingly, there was no positive signal when anti-MICA was used as capture antibody and either of the anti-tetraspanins (CD9 or CD63) antibodies were used for detection. This was also true when CD63 or CD81-specific antibodies were used for capture and anti-MICA for detection. Since these results differed markedly from the results of the ELISA platform, in which all combinations gave a positive result, we inferred that antibody recognition of the epitope was not a problem. We hypothesized that incubation of the different antibodies in solution in the first step of LFIA could generate steric hindrance affecting the outcome of the experiment, as we had previously speculated [25].

**Table 1:** Combination of antibodies used for LFIA

Detection antibody	Capture antibody			
	MICA	CD63	CD81	CD9
MICA	ND	–	–	+
CD63	–	ND	25	25
CD9	–	–	25	ND

25 corresponds to the reference list (Oliveria-Rodríguez et al. [25])

ND, not determined; −, negative signal; +, positive signal

Since further optimization of the LFIA platform for exosome recognition of proteins was required, we assayed the effects of various modifications to the protocol, including the addition of different concentrations of the detergent Tween-20 or ethanol to the running buffer, the use of increasing concentrations of anti-MICA antibody, the inclusion of a pre-incubation step of detection antibody to exosomes and performing the assay in two steps (first running the exosomes and then the detection antibody) (Additional file 1: Figure 1B, C, D). These experiments defined the optimal basic assay conditions: using 0.05% Tween-20 in running buffer, 20 µL of AuNP coupled to anti-MICA detection antibody (prepared as explained in the “Methods” section) and pre-incubating exosomes with detection antibody for 1 h.

**Fig. 3:**
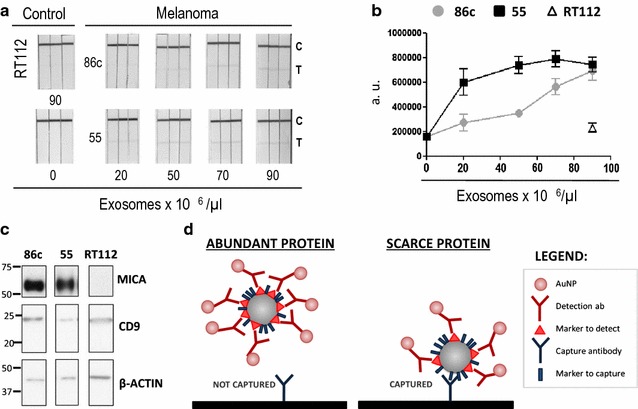
Exosomal MICA detected by LFIA from different melanoma cell lines. **a** Scanned LFIA strips of the exosome titration. Melanoma exosomes were derived from either Ma-Mel-86c (86c) or Ma-Mel-55 (55), incubated with AuNP-anti-MICA, as detection antibody, in a final volume of 100 μL and run for 15 min in triplicates in strips containing a test line of anti-CD9. Two negative controls were included: a sample containing 90 × 10^6^ exosomes from the bladder cancer line RT-112 which does not express detectable MICA and a sample without exosomes (buffer only). The positions of the test (T) and control lines (C) are indicated. **b** Electronic quantitation of the detected signal. The Test line signal from A was quantified using the ChemiDoc™ MP Imaging System and represented as arbitrary units (a.u.). Data are the mean and SEM of the triplicates. **c** Comparison of MICA levels by Western blot. The relative amounts of MICA and CD9 expression were checked in the same sample used for LFIA: melanoma exosomes derived from Ma-Mel-86c (86c), Ma-Mel-55 (55) and the bladder cancer cell line RT-112. Actin was used as loading control. **d** Model proposed. Based on the results of the LFIA experiments comparing exosomes containing different quantities of the detected marker we propose a steric hindrance model: if a marker is abundant, the exosome is completely covered by detection antibody. This reduces the availability of epitopes for the capture antibody (left panel). However, if the detection marker is more scarce, the exosome can be captured (right panel)

Next, to define the specificity, limits of detection and saturation properties of the assay, the amounts of exosomes from two different metastatic melanoma cell lines, Ma-Mel-86c and Ma-Mel-55, were titrated (Fig. 3a). The signal was quantified using a non-biased electronic tool and saturation curves were obtained (Fig. 3b). As a control of the assay specificity, a high concentration of exosomes from the bladder cell line RT112, devoid of MICA (Fig. 3c) were assayed in parallel producing no signal on the test line (depicted as a triangle in Fig. 3b). Surprisingly, quantitative analysis of the MICA-containing exosomes from the melanomas yielded some interesting results: the signal from Ma-Mel-55 exosomes was higher than that of Ma-Mel-86c exosomes and reached a plateau at lower exosome concentration (Fig. 3b), even though the amounts of MICA and CD9 protein in the exosome preparation from Ma-Mel-55 were lower than in Ma-Mel-86c, when compared by Western blot (Fig. 3c). This observation, together with the fact that several combinations of antibodies that worked well in ELISA were less efficient for detection in LFIA, led us to hypothesize that if the protein being detected is very abundant, the exosomes may become completely covered by the detection antibody (always used in excess so that it is available for capture in the control line, for flow validation of the test, after binding to the antigen). In these circumstances, steric hindrance probably makes the second epitope of the exosome inaccessible for capture in the test line (Fig. 3d). This model is also supported by data obtained using as detection antibody CD9 in combination with either CD63 or CD81 for capture (Data not shown and [25]).

### Optimization of LFIA for protein detection on exosomes requires low antibody concentration in the gold nanoparticles

**Fig. 4:**
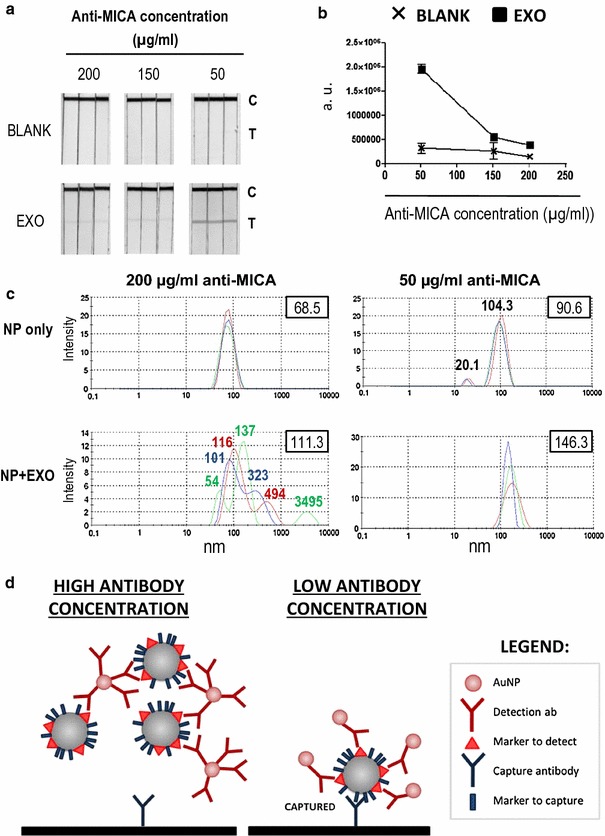
Low concentration of antibody in the AuNP increases exosome detection by LFIA. **a** Scanned LFIA strips. AuNP coupled to decreasing amounts of anti-MICA antibody were prepared and used for detection. Either 50 × 10^6^/µL Ma-Mel-86c exosomes (EXO) or no exosomes as control (BLANK) were run in strips containing a test line of anti-CD9. The position of the test (T) and control lines (C) is indicated in figure. **b** Electronic quantitation of the detected signal. Signal in the test line from **a** was quantified using a ChemiDoc™ MP Imaging System and represented as arbitrary units (a.u.). Data represent the mean and SEM of the triplicates. **c** Hydrodynamic diameter distribution. DLS analysis of AuNP conjugated to different amounts of anti-MICA antibody incubated either alone or with 50 × 10^6^/µL of melanoma-derived exosomes. The graph shows three independent readings of each sample. The value for each peak and the average value (upper right corner) are indicated in the graph. **d** Model of the effect for exosome detection of the antibody concentration coupled to AuNP. When gold nanoparticles are conjugated using high concentration of antibody, higher order aggregates are generated due to the crosslinking of antibodies and exosomes. This results in an impaired flow of the mixture on the strip and lower capture capacity (left panel). However, if the detection antibody is conjugated at limiting concentration, these complexes do not form and each exosome can bind to several AuNP, resulting in better signal detection (right panel)

The unexpected results with exosomes containing different amounts of MICA, apparently due to steric issues and the observation that the sample pads acquired colour from the gold nanoparticles, increasing with the pre-incubation time and with the number of exosomes (Additional file 1: Figure 2A, B), prompted new experiments to check whether the evaluation of exosomes by LFIA was affected by the formation of nanoparticle complexes which could alter the flow of the exosome sample in the strip. For this purpose, the concentration of anti-MICA antibody conjugated to AuNP was reduced, trying to couple four times less antibody per AuNP. NP with low amounts of coupled antibody were then compared with NP with the maximal antibody conjugation (Fig. 4a, b). Interestingly, MICA was detected seven times better when AuNP with limited amounts of antibody on their surface were used. Moreover, analysis of the hydrodynamic diameter using Dynamic Light Scattering (DLS) revealed that, if AuNP were saturated with antibody, the incubation with exosomes results in several peaks corresponding to aggregates of different sizes, while AuNP with limiting amounts of antibody displayed a uniform peak, with no evidence of aggregate formation (Fig. 4c).

Taken together, these results indicate that in order to develop LFIA for proteins in exosomes, it is necessary to consider the relative amounts of the proteins within the exosome as well as the multimeric nature of exosomes, which can lead to aggregation of AuNP, because several epitopes for each antibody are present in each vesicle. Thus, this technique requires a careful optimization to deal with the possibility of formation of AuNP-exosome complexes of different sizes, leading to altered diffusion of the sample and a reduction in the signal detected (Fig. 4d).

### MICA can be detected using LFIA in human serum

**Fig. 5:**
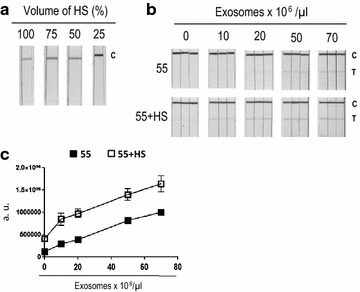
Exosomal MICA detection in human serum by LFIA. **a** Optimal human serum dilution in running buffer. Different volumes of exosome-depleted human serum were run with anti-MICA detection antibody-conjugated NP, but no exosomes, to identify background signal. **b** Titration of exosomes for MICA detection in human serum by LFIA. Melanoma exosomes derived from Ma-Mel-55 (55) were tested without or with 25% of exosome-depleted human serum added to running buffer (55 + HS). The mixture was run in the strips using anti-CD9 as capture antibody. Scanned strips of the experiment in triplicates are shown. The position of the test (T) and control lines (C) is indicated in figure. **c** Quantitation of the detected signal. Signal in the test line from B was quantified using the ChemiDoc™ MP imaging system and represented as arbitrary units (a.u.). Data represent the mean and SEM of the triplicates

Since it is well established that NKG2D-ligands are found in high levels in the serum of different types of cancer patients, and the ultimate goal of developing new tools for detection of MICA in exosomes is the analysis of biological fluids, we evaluated the technique replacing running buffer with increasing amounts of human serum. The effect of viscosity from exosome-depleted human serum was first evaluated running antibody-only samples in the assay. As expected, the baseline signal detected in the control line increased. The best results were obtained when serum was diluted 1:4 in running buffer (Fig. 5a), indicating that the presence of detergent increases the fluidity of the sample and increases the signal/noise ratio. The titration of melanoma-derived exosomes in serum compared to buffer revealed that the signal was higher, due to an increase in the background measured in the negative control (Fig. 5b, c). Although it is likely that high concentration of salts and ions present in sera can cause interference [27], these data show that positive signals for similar concentration of exosomes can be detected in either serum or buffer-based samples.

## Discussion

In this study we have optimized two methods, ELISA and LFIA, for the detection in exosomes of MICA, a protein released from cancer cells that can affect immune recognition mediated by the activating receptor NKG2D. Both techniques have the advantage that they can be used as high throughput assays and thus, could be used in clinical studies. Also, both techniques allow detection of two different epitopes and so, they prove the co-existence in the same vesicle of an exosomal marker and a particular antigen. As well as describing the proof-of-concept for the simultaneous detection of MICA and tetraspanins in exosomes, these data also reveal important differences in the use of these techniques for detection of proteins in exosomes versus soluble molecules. Further, we have identified important features that must be taken into consideration when optimising LFIA for exosomal protein detection to avoid misleading aggregation or steric effects. In fact, we demonstrate here that, in LFIA, conjugating fewer antibodies per AuNP, unexpectedly, results in better sensitivity for exosome detection.

We also demonstrate here for the first time that MICA is contained in the same vesicle as the tetraspanins CD9, CD63 and CD81. We have previously described that MICA*008 can be recruited to exosomes and that it migrates in the same sucrose gradient fractions in which exosomes were detected (tetraspanin positive fractions by Western blot) [20]. We proved that vesicles can be captured binding a MICA epitope and this allows detection of several tetraspanins in the same vesicle. Further, we use a model in which MICA protein is expressed endogenously in vesicles secreted by melanoma cell lines and demonstrate that they are functionally active, down-modulating the receptor NKG2D on lymphocytes. Thus, we have been able to perform an exhaustive characterization of MICA-containing exosomes that suggests the important role that the secretion of these proteins in exosomes can have in vivo. The release of MICA-containing exosomes to serum will depend on the allele present in each individual’s genome and it would be desirable to perform broader analyses of patients including the detection of immune modulating exosomes.

**Fig. 6:**
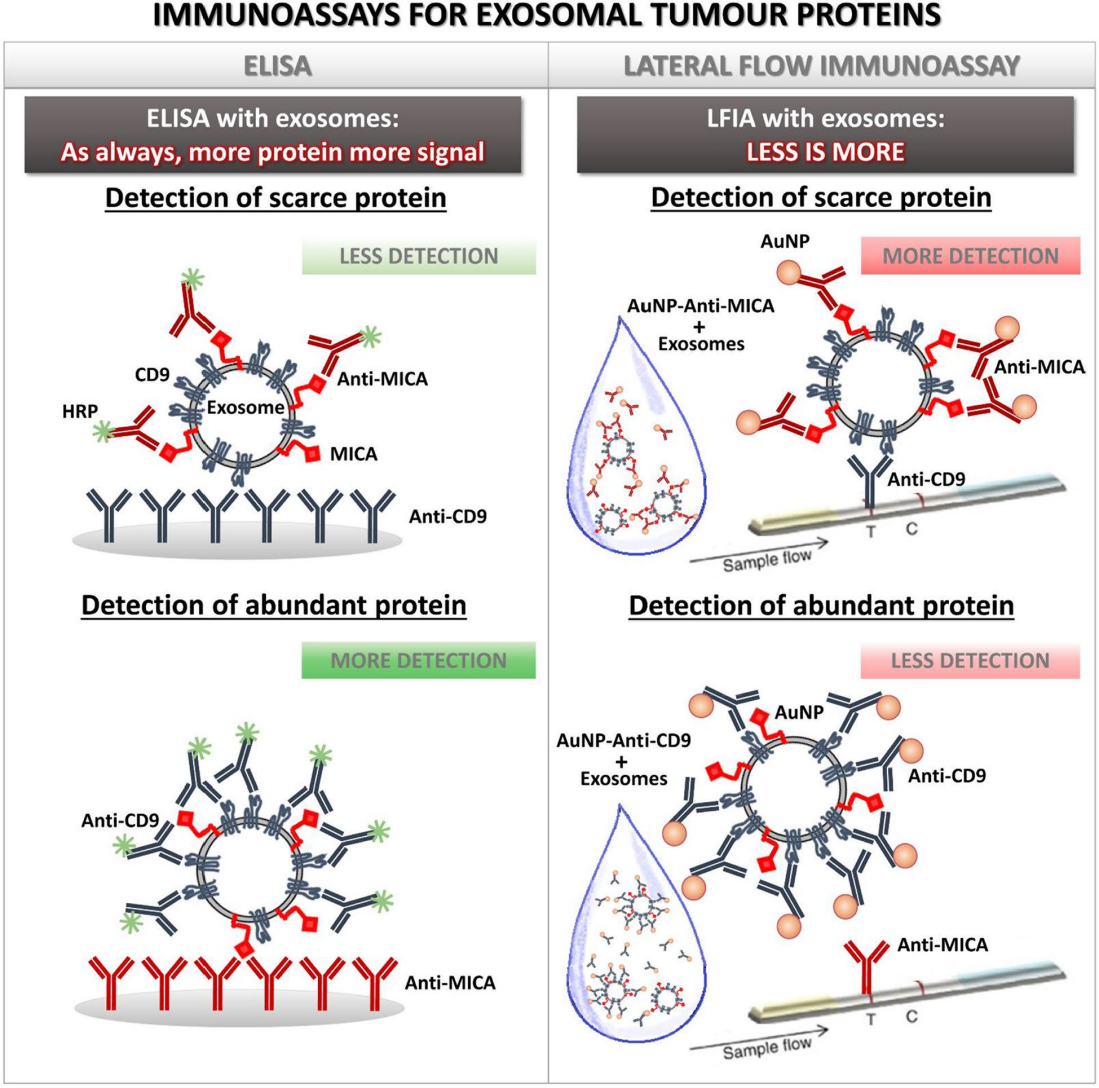
Immunocapture of proteins on exosomes requires careful optimization. Although most immunodetection techniques share a common basis, i.e. binding with a capture antibody and detection with a complementary antibody, the methodological procedure affects the ability of a reagent to bind to the sample. In ELISA, incubation with the detection antibody is done after capture of exosomes on the antibody-coated plated while, in LFIA, exosomes are mixed in solution with the antibody. Thus, in ELISA, one side of the exosome is available for binding while, in LFIA, the exosome can be completely recovered by detection antibody. As a consequence, in ELISA, more detection antibody bound to the exosome results in a higher signal while in LFIA, more detection antibody, results in steric hindrance and the impossibility of being captured

It is widely known that any technique used for a new purpose requires optimization and here we demonstrate that, for detection of proteins on exosomes, the combination of capture and detection antibodies chosen significantly influences the outcome of each method. In ELISA, the abundance of an epitope in an exosome does not affect binding of the second antibody, because one side of the vesicle faces the coated plate leaving the other side of the vesicle available for binding. In contrast, in LFIA, the abundance of the epitope can impair binding of the second antibody, because that first incubation between antibody and exosomes occurs in solution, and thus, the detection antibody may bind over the entire surface of the vesicle (Fig. 6). For this reason, if the detection antibody is directed against a very abundant epitope on the exosome, steric impediments with the capture antibody are more likely to occur. Comparing two types of exosomes containing different amounts of MICA, as demonstrated by Western blot, our data also prove that the amount of the protein detected in exosomes is not necessarily linear with respect to the signal obtained. In these experiments, it was possible to detect MICA more efficiently (higher absolute reading and faster slope and saturation curve) when the epitope was less abundant. This was also observed when trying different combinations of tetraspanins [25]. Further, steric issues are also an important factor to be considered when detecting exosomes by LFIA, since the existence of several epitopes in the same vesicle and the coupling of multiple detection antibodies per AuNP can result in the formation of large masses of aggregates. Thus, we recommend optimizing coating of detection antibody to AuNP in a non-saturating concentration range, since the signal can be later enhanced by immobilising in the test line a capture antibody directed against abundant tetraspanins, like CD9 in our melanoma cell model.

With the results from this study, we can compare the limits for the detection of MICA using ELISA and LFIA. However, it is important to stress that, while in ELISA the signal obtained is directly proportional to the amount of epitopes per exosome, for LFIA, more MICA per exosome translated into lower signal, so this technique has to be optimized with care for qualitative analysis and complemented with a quantitative technique. In both cases, MICA-containing exosomes were easily detected in a concentration ranging around 5 × 10^7^ exosome particles per μl. The last aspects to compare on the two techniques used here are the time consumed for assay implementation and price per sample analysed; as usual, LFIA is fast and relatively cheaper than ELISA. Thus, depending on the final application either method can be of great interest to implement for either basic or clinical research of exosomal surface proteins.

It is widely known that the presence of plasma can increase the background noise in ELISA. Thus, we evaluated the effect of plasma when making the measurements by LFIA. As in other methods, plasma increased the signal in control samples, however, positive samples also had a clear increase. Thus, we can anticipate that LFIA could be used to evaluate the presence of particular proteins in exosomes from patient samples.

The requirement of non-abundant epitopes for detection represents an advantage for LFIA; tumour-derived antigens are, in general, much less abundant than tetraspanins in exosomes [3] and, in biological fluids, tumour-derived exosomes are expected to be a minority in plasma, since they will be mixed with exosomes from healthy cells. This technique permits the enrichment of tumour derived-exosomes and enhanced visualization with an abundant tetraspanin marker.

In conclusion, in this paper we have described the detection of the non-abundant tumour-derived antigen MICA in exosomes using two immunodetection-based methods and we describe the different parameters that require careful optimization for the implementation of new exosome markers. Strikingly, antibody coating of AuNP needs to be titrated down for a better detection in LFIA.

## Methods

### Reagents and cells

Reagents were purchased from sigma, unless otherwise stated.

The human metastatic melanoma cell lines Ma-Mel-55 and Ma-Mel-86c were previously described [26], and genotyped for MICA. Both cell lines only express MICA A5.1, which is recruited to exosomes. Cells were grown in RPMI-1640 supplemented with 10% FCS, 1 mM glutamine, 1 mM sodium pyruvate, 0.1 mM nonessential amino acids, 10 mM Hepes, 100 U/mL penicillin and 100 U/mL streptomycin (Biowest). The bladder cancer cell line RT-112 is available from the ATCC [kindly provided by Dr. FX Real (CNIO, Madrid) and genotyped using the StemElite ID System (Promega) at the Genomics Service (IIB-CSIC)]. These cells were grown in EMEM medium (Lonza) supplemented with 10% FCS, 1 mM glutamine, 1 mM sodium pyruvate, 0.1 mM non-essential amino acids, 100 U/mL penicillin, and 100 U/mL streptomycin (Biowest).

Peripheral blood mononuclear cells (PBMC) were purified from healthy volunteer buffy coats (Regional Transfusion Centre, Madrid). Approval from local ethical committees (Transfusion Centre and CSIC) and informed consent from all participants were obtained. After Ficoll isolation, PBMCs were stimulated with 10 U/mL IL-12 (Peprotech) and 25 mg/mL IL-18 (MBL) to allow enrichment of the Natural Killer (NK) cell population. Buffy coats (Regional Transfusion Centre, Madrid) were used under approval from local ethical committees (Transfusion Centre and CSIC) and informed consent from all participants.

### Exosome isolation, quantitation and EM visualization

For exosome enrichment, cells were cultured for 3–5 days in their appropriate medium, but with 1% exosome-free FCS (prepared by centrifugation at 100,000×*g* for 18 h and sterile-filtered). Cell culture supernatants were centrifuged for 10 min at 200×*g* to discard cells and exosomes were purified by sequential centrifugation as previously described [20, 21]. Briefly, supernatants were centrifuged for 10 min at 500×*g*, 30 min at 10,000×*g* and finally ultracentrifuged at 100,000×*g* for 2 h at 4 °C (Beckman Instruments). The pellet, containing exosomes, was resuspended in HEPES-buffered saline buffer (HBS: 10 mM HEPES pH 7.2, 150 mM NaCl). For liophylization the buffer was supplemented with 8% sucrose, samples were frozen immediately at − 80 °C and lyophilized using a Flexi-Dry Lyophilizer (FTS Systems).

Exosome concentration was determined by nanoparticle tracking analysis (NTA) in a NanoSight NS500 (Malvern Instruments Ltd, Malvern, UK). A 405-nm laser beam was used to highlight the particles, which act as point scatters. Analysis was performed using the NTA 3.1 software (Malvern). These experiments were carried out at the laboratory of Dr. H. Peinado, Spanish National Centre for Oncological Research (CNIO). Electron microscope examination of exosomes was carried out as previously described [25].

### Western blot

The same number of purified exosomes (7 × 10^9^) resuspended in Laemmli buffer (non-reducing condition for tetraspanins) was run on 12% SDS-PAGE gels. Samples were transferred to Immobilon-P (Millipore) membrane. The membrane was blocked using PBS containing 0.1% Tween-20 (PBS-T) and 5% non-fat dry milk. The tetraspanins, CD63, CD9 and CD81, were detected by incubation with MEM-63, MEM-9 and MEM-81, respectively [kind gift from Vaclav Horejsi (Czech Republic)]. For MICA detection, the membrane was incubated with biotinylated goat polyclonal anti-MICA antibody (R&D) (BAF1300). Mouse anti- β-actin (AC-15) was from Sigma. The secondary antibody goat anti-mouse Ig-HRP (Dako) or streptavidin-HRP (Biolegend) were used before developing using the ECL system (GE Pharmaceuticals).

### NKG2D downmodulation

The day before the experiments, PBMC were stimulated with 20 ng/mL IL-15 (Peprotech) for 18 h. The percentage of NK cells in PBMC cultures was assessed by flow cytometry using CD3 and CD56-specific mAbs. PBMC (10^5^ NK cells) were incubated in 96-well flat-bottomed plates with 1–5 × 10^10^ melanoma-derived exosomes (or with exosome-free RPMI medium, as negative control). After 24 h, NKG2D surface expression on NK cells was monitored by staining with PE-conjugated anti-NKG2D antibody, FITC-anti-CD3 (BioLegend) and APC-anti-CD56 (e-Bioscience), and flow cytometry analysis using the cytometer Cytomics FC 500 (Beckman Coulter) and Kaluza (Beckman Coulter) software.

### ELISA

MICA and tetraspanins were detected in melanoma-derived exosomes using sandwich ELISA. Plates were coated with purified mAb R&D against MICA (MAB13002) or anti-tetraspanin antibodies (anti-CD9 VJ1/20 [28] or anti-CD63 Tea3/18 [29] in BBS (Borate Buffered saline) overnight at 4 °C. After blocking the plates with 2% BSA-HBS for 2 h at 37 °C, melanoma-derived exosomes were added (or HBS as negative control) and incubated overnight at 4 °C. Biotinylated secondary antibodies [CD9 VJ1/20 [25] and MICA (BAF1300, R&D)] were added and followed by streptavidin-HRP (Amersham). The reaction was developed using the peroxidase substrate system (ABTS; Roche). Absorbance was measured at 405 nm with a reference wavelength of 490 nm with Thermo Scientific™ Multiskan™ FC Filter-based Microplate Photometer (Thermo Fisher Scientific).

### Size distribution

Size distribution was measured with a Zetasizer Nano ZS ZEN3600 (Malvern Instruments, Malvern, UK). The samples were diluted (50- to 100-fold) fractions and 3 readings were performed. Each reading was composed of 15 measurements of the backscattered (173°) intensity. Zetasizer software version 7.03 was used for data processing and analysis.

### Lateral flow immunoassay

The LFIA was carried out in a dipstick format as described in [25]. Capture antibodies [anti-MICA (1H10), anti-CD63 (Tea3/18), anti-CD81 (5A6, provided by Dr. S. Levy, Department of Oncology, Stanford University School of Medicine, Stanford, CA), anti-CD9 (VJ1/20, Immunostep)] and control anti-IgG antibody were loaded onto a nitrocellulose membrane to make a test and a control line in the strip, using a dispenser IsoFlow (Imagene Technology, USA) at a flow rate of 0.100 µL/mm. For trial experiments, the lines were prepared manually, dispensing 3 × 0.3 µL of the corresponding antibody (1 mg/mL). The membrane was dried for 20 min at 37 °C.

Detection antibodies [anti-CD9 (VJ1/20), anti-CD63 (Tea3/18), anti-MICA (1H10, [30] or MAB13002 from R&D)] were conjugated to 40 nm gold nanoparticles (AuNP) from BB International (UK). A gold colloid titration was done to estimate the optimal concentration of the antibody to stabilize the gold nanoparticles [31]. For anti-CD63 and anti-MICA labelling, a non-covalent bioconjugation protocol was followed, while a covalent procedure was necessary for anti-CD9, protocols previously described in [25].

Purified exosomes were prepared in running buffer (10 mM HEPES, pH 7.4, with 150 mM NaCl, 0.05% Tween-20 and 1% BSA) and transferred to a microtube containing 20 µL of antibody-coupled AuNP (in the experiments shown in Additional file 1: Figure 1A and B, 10 µL) in a final volume of 100 µL. For negative control, buffer without exosomes was added to the detection antibody. For most of the experiments, a pre-incubation of 1 h was used prior to introduction of the dipstick in the microtube for a run of the LFIA of 15 min. 100 µL of running buffer were added after the sample. When other pre-incubation times were used, it is indicated in figure legend.

After the run, the signal obtained in the test line was quantitated by scanning the strips using ChemiDoc™ MP Imaging System and analyzing with Image Lab Software (Bio-Rad).

### Stastistical analysis

Student’s t-test was done for paired samples using GraphPad Prism 5. P-values are shown in each figure; only significant differences are indicated.

## Additional file

**Additional file 1: Figure S1.** Optimization of LFIA for exosomal MICA detection. A. Antibodies combination. Different antibody combinations for capture and detection, as indicated, were tried. B. Running buffer composition. The addition of different concentrations of Tween 20 and ethanol with anti-MICA in the running buffer was tested. In A and B, the amount of antibody-coupled AuNP was 10 µL, thus, for comparison, the same set of strips was used in the no ethanol (0% ethanol) and 10 µL of detection antibody sets. C. Incubation time. Melanoma exosomes were incubated with the detection antibody anti-MICA conjugated to AuNP for different times prior to the run on the strip. D. Effect of including two steps for dispensing exosomes and antibody-coupled NP to the LFIA strip. Exosomes were incubated with detection anti-MICA antibody NP and dispensed to the dipstick in one step. Alternatively, exosomes were run first and, in a second step, the detection antibody-coupled NP. The test strips in triplicates are shown. Right graph: quantitation of the test line signal represented as arbitrary units (a.u.). Data are the mean and SEM of the triplicates. The capture antibodies were immobilized manually in the strips and exosomes were not pre-incubated with detection antibody for one hour in A and B. In B, C and D, anti-CD9 was used as capture antibody and anti-MICA-NP for detection. Melanoma exosomes derived from Ma-Mel-55 (55) or Ma-Mel-86c (86c) were run (E, EXO) or no exosomes as control (B, BLANK). The position of the test (T) and control lines (C) are depicted. **Figure S2.** Visual analysis of the sample pads colour. Pads from the experiments above were scanned to appreciate the material still embedded in the pad at the end of the running time. A. Effect of incubation time. Pads from the experiments shown in Additional Figure 1C. B. Titration of exosome concentration. Pads from the experiments shown in Figure 4B.

## Abbreviations

BBS: borate buffered saline
DLS: dynamic light scattering
ELISA: enzyme-linked immunosorbent assay
EVs: extracellular vesicles
AuNP: gold nanoparticles
HBS: HEPES-buffered saline buffer
HRP: horse radish peroxidase
LFIA: lateral flow immunoassays
MICA: MHC class I chain-related protein A
NTA: nanoparticle tracking analysis
NK: natural killer
NKG2D: natural killer group 2D
PBMC: peripheral blood mononuclear cells
