# Supplementary material for: Immunoassays for scarce tumour-antigens in exosomes: detection of the human NKG2D-Ligand, MICA, in tetraspanin-containing nanovesicles from melanoma
Source: J Nanobiotechnology. 2018 May 2;16:47. doi: 10.1186/s12951-018-0372-z (PMC5932892; doi:10.1186/s12951-018-0372-z)
Supplement: Supplementary file 1 — Additional file 1: Figure S1. Optimization of LFIA for exosomal MICA detection. A. Antibodies combination. Different antibody combinations for capture and detection, as indicated, were tried. B. Running buffer composition. The addition of different concentrations of Tween 20 and ethanol with anti-MICA in the running buffer was tested. In A and B, the amount of antibody-coupled AuNP was 10 µL, thus, for comparison, the same set of strips was used in the no ethanol (0% ethanol) and 10 µL of detection antibody sets. C. Incubation time. Melanoma exosomes were incubated with the detection antibody anti-MICA conjugated to AuNP for different times prior to the run on the strip. D. Effect of including two steps for dispensing exosomes and antibody-coupled NP to the LFIA strip. Exosomes were incubated with detection anti-MICA antibody NP and dispensed to the dipstick in one step. Alternatively, exosomes were run first and, in a second step, the detection antibody-coupled NP. The test strips in triplicates are shown. Right graph: quantitation of the test line signal represented as arbitrary units (a.u.). Data are the mean and SEM of the triplicates. The capture antibodies were immobilized manually in the strips and exosomes were not pre-incubated with detection antibody for one hour in A and B. In B, C and D, anti-CD9 was used as capture antibody and anti-MICA-NP for detection. Melanoma exosomes derived from Ma-Mel-55 (55) or Ma-Mel-86c (86c) were run (E, EXO) or no exosomes as control (B, BLANK). The position of the test (T) and control lines (C) are depicted. Figure S2. Visual analysis of the sample pads colour. Pads from the experiments above were scanned to appreciate the material still embedded in the pad at the end of the running time. A. Effect of incubation time. Pads from the experiments shown in Additional Figure 1C. B. Titration of exosome concentration. Pads from the experiments shown in Figure 4B. [file 12951_2018_372_MOESM1_ESM.pdf]

Additional Figure 1

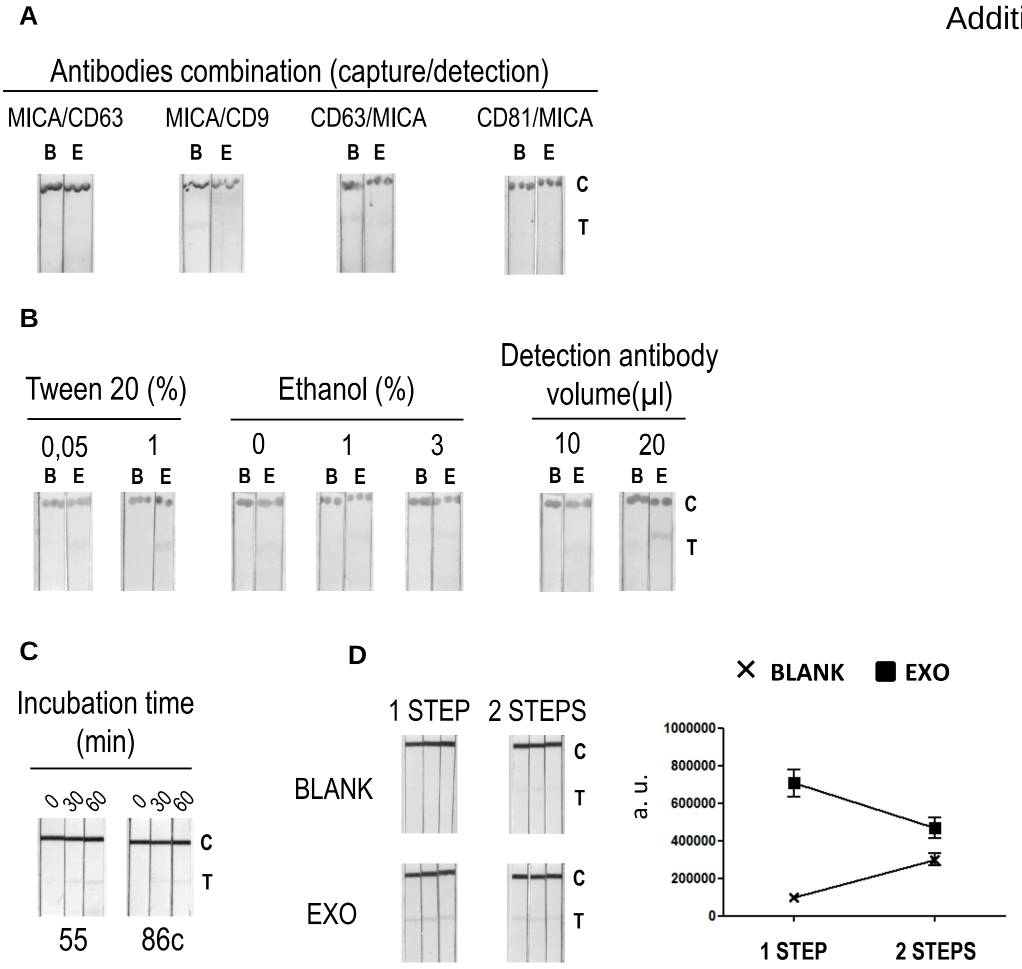

Additional Figure 1. Optimization of LFIA for exosomal MICA detection. A. Antibodies combination. Different antibody combinations for capture and detection, as indicated, were tried. **B. Running buffer composition.** The addition of different concentrations of Tween 20 and ethanol with anti-MICA in the running buffer was tested. In A and B, the amount of antibody-coupled AuNP was 10 µl, thus, for comparison, the same set of strips was used in the no ethanol (0% ethanol) and 10 µl of detection antibody sets. **C. Incubation time.** Melanoma exosomes were incubated with the detection antibody anti-MICA conjugated to AuNP for different times prior to the run on the strip. **D. Effect of including two steps for dispensing exosomes and antibody-coupled NP to the LFIA strip.** Exosomes were incubated with detection anti-MICA antibody NP and dispensed to the dipstick in one step. Alternatively, exosomes were run first and, in a second step, the detection antibody-coupled NP. The test strips in triplicates are shown. Right graph: quantitation of the test line signal represented as arbitrary units (a.u.). Data are the mean and SEM of the triplicates. The capture antibodies were immobilized manually in the strips and exosomes were not pre-incubated with detection antibody for one hour in A and B. In B,C and D, anti-CD9 was used as capture antibody and anti-MICA-NP for detection. Melanoma exosomes derived from Ma-Mel-55 (55) or Ma-Mel-86c (86c) were run (E, EXO) or no exosomes as control (B, BLANK). The position of the test (T) and control lines (C) are depicted.

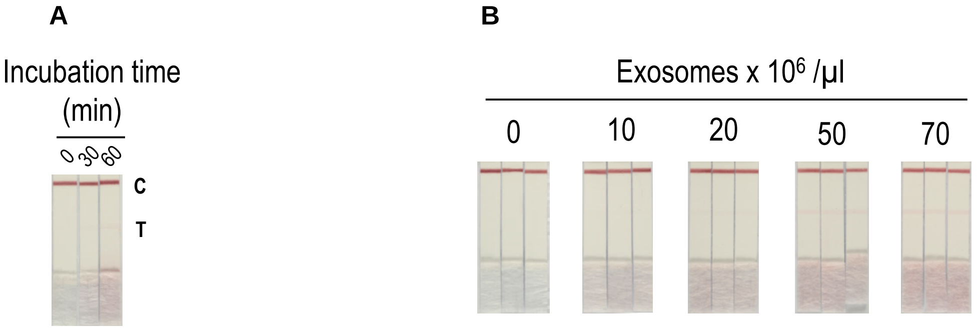

Additional Figure 2. Visual analysis of the sample pads colour. Pads from the experiments above were scanned to appreciate the material still embedded in the pad at the end of the running time. **A. Effect of incubation time.** Pads from the experiments shown in Supplementary Figure 1C. **B. Titration of exosome concentration.** Pads from the experiments shown in Figure 4B.
